# Osteoponin Promoter Controlled by DNA Methylation: Aberrant Methylation in Cloned Porcine Genome

**DOI:** 10.1155/2014/327538

**Published:** 2014-07-02

**Authors:** Chih-Jie Shen, Yung-An Tsou, Hsiao-Ling Chen, Hung-Jin Huang, Shinn-Chih Wu, Winston T. K. Cheng, Calvin Yu-Chian Chen, Chuan-Mu Chen

**Affiliations:** ^1^Department of Life Sciences, National Chung Hsing University, Taichung 402, Taiwan; ^2^School of Medicine, College of Medicine, China Medical University, Taichung 40402, Taiwan; ^3^Department of Otolaryngology Head and Neck Surgery, China Medical University, Taichung 40402, Taiwan; ^4^Department of Bioresources, Da-Yeh University, Changhwa 515, Taiwan; ^5^Department of Chinese Pharmaceutical Sciences and Chinese Medicine Resources, College of Pharmacy, China Medical University, Taichung 40402, Taiwan; ^6^Department of Animal Science and Technology, National Taiwan University, Taipei 106, Taiwan; ^7^Department of Animal Science and Biotechnology, Tung Hai University, Taichung 407, Taiwan; ^8^Department of Biomedical Informatics, Asia University, Taichung 41354, Taiwan; ^9^Department of Medical Research, Human Genetic Center, China Medical University Hospital, Taichung 40447, Taiwan; ^10^Research Center for Chinese Medicine & Acupuncture, China Medical University, Taichung 40402, Taiwan; ^11^Rong Hsing Research Center for Translational Medicine and the iEGG Center, National Chung Hsing University, Taichung 402, Taiwan

## Abstract

Cloned animals usually exhibited many defects in physical characteristics or aberrant epigenetic reprogramming, especially in some important organ development. Osteoponin (OPN) is an extracellular-matrix protein involved in heart and bone development and diseases. In this study, we investigated the correlation between *OPN* mRNA and its promoter methylation changes by the 5-aza-dc treatment in fibroblast cell and promoter assay. Aberrant methylation of porcine *OPN* was frequently found in different tissues of somatic nuclear transferred cloning pigs, and bisulfite sequence data suggested that the *OPN* promoter region −2615 to −2239 nucleotides (nt) may be a crucial regulation DNA element. In pig ear fibroblast cell culture study, the demethylation of *OPN* promoter was found in dose-dependent response of 5-aza-dc treatment and followed the *OPN* mRNA reexpression. In cloned pig study, discrepant expression pattern was identified in several cloned pig tissues, especially in brain, heart, and ear. Promoter assay data revealed that four methylated CpG sites presenting in the −2615 to −2239 nt region cause significant downregulation of *OPN* promoter activity. These data suggested that methylation in the *OPN* promoter plays a crucial role in the regulation of *OPN* expression that we found in cloned pigs genome.

## 1. Introduction

Nowadays, many of pathogenesis of diseases have been determined [1–3]. Methylation in the 5′ cytosine in the CpG dinucleotides is crucial a mechanism that regulates gene expression without changing DNA sequence and can be inherited to the offspring [4]. The promoter region contains various transcription factor binding motifs with numerous CpG dinucleotides. Some transcription factors are blocked by methylated CpG island resulting in inhibition of gene expression [5]. Somatic cell nuclear transfer (SCNT) technique is used to generate an identical genetic background offspring [6, 7]. However, SCNT cloning animals usually showed low survival rate and impropriate methylation reprogramming process [8]. This dilemma of SCNT animal may be caused by methylation controlled genes, such as imprinting genes [9].

*OPN* is an extracellular matrix protein and hydrophilic glycoprotein identified firstly in the bone as a sialoprotein. It contains a thrombin and transglutaminase cutting site, and the molecular weight is about 25 kDa to 75 kDa; in pig, the molecular is about 67 kDa; it contains numerous isoforms [10].* OPN* has a hydrophobic N terminal; thus, it can be secreted out of cell membrane; the amino sequence of* OPN* is full of Asp, Thr, and Ser that can elevate the binding activity with calcium, glycosylation, and phosphorylation, respectively [11]. Thus,* OPN* plays numerous roles in many aspects, such as bone remodeling, cell migration, iNOS regulation, repairment, and leucocyte recruitment [12]. And acquired* OPN* expression has been found in a variety of cancer cell types, especially in the liver, lung, breast, prostate, colon, brain, and spleen [13, 14].* OPN* is cleaved by MMPs protein to generate functional* OPN* that can bind to *α*v*β*3 [15]. This integrin binding with* OPN* has influence on NF*κ*B signaling transduction [16, 17]. Therefore, overexpressed* OPN* is associated with tumorigenesis, tumor invasion, and metastasis [18, 19]. Previous study suggested that overexpressed* OPN* induces the serious cardiac fibrosis [20, 21]. Thus, our cloned pigs were also surrounded by various defects in heart fibrosis and retardation of growth of bones. Therefore, this study focuses on the methylation change of* OPN* promoter that may be disrupted by inappropriate reprogramming process. Consequently, aberrant methylation of promoter could lead to aberrant expression of* OPN*. In the previous studies,* OPN* expression was induced with TSA (trichostatin A) in mouse undifferentiated mesenchymal cell line by AP1 site [22]. The TSA is a histone deacetylase inhibitor. It can lose the chromatin structure in order to let gene restore its expression. 5-aza-dc is also an analog with the same structure of cytosine without methyl group adding in the 5′C end [23].

Thus, 5-aza-dc addition leads to low methylation percentage in the CpG sites rich region. The hypomethylation status in the promoter may contribute its gene transcription activity. Porcine fetal fibroblasts in 5th passage cultures were treated with 0.5, 1.0, 2.0, and 3.0 *μ*M 5-aza-dc for 96 h; 5-aza-dc inhibited the growth of cell at all concentrations. 5-aza-dc induced a reduction of transcripts level in* DNMT1* and increasing expression in imprinted gene,* IGF2 *[24]. Furthermore human* OPN* promoter sequence is similar to porcine in the front 400 nt of the porcine promoter. Therefore, we investigated* OPN* RNA and promoter methylation changes in the porcine ear fibroblast cell. Data showed that the elevated* OPN* expression and in 5-aza-dc treated fibroblast cell is due to the decreased methylation of* OPN* promoter. Cloned pigs samples had found extremely methylation changes, especially in the brain (99.75% upregulation), heart (11.50% down-regulation), and ear (18.03% down-regulation). Deletion analysis of the promoter region revealed 5-aza-dc induced luciferase response that was regulated by −2615 to −2239 of the* OPN* promoter. These data suggested that methylation in the* OPN* promoter plays a crucial role in the regulation of* OPN* expression. Methylation of* OPN* promoter may be an epigenetic marker of diagnosis of cancer.

## 2. Materials and Methods

### 2.1. CpG Island Prediction

The sequence of a putative CpG island in* OPN *promoter was analysed by using MethPrimer software (http://www.urogene.org/methprimer/index1.html).

### 2.2. Cell Culture

The porcine fibroblast cell line was grown in Dulbecco's modified Eagle's medium (DMEM; Gibco-BRL, Gaithersburg, MD, USA) supplemented with 10% fetal bovine serum (FBS, Gibco BRL) and containing 100 U/mL penicillin and streptomycin. The cells were incubated at 37°C in humidified incubator with 5% CO_2_.

### 2.3. 5-aza-dc Demethylation Drug Treatment

For 5-aza-dc treatment, porcine fibroblast cell in 5th passage cultures was treated with 5-aza-dc (sigma) at various concentrations, that is, 0 (control), 0.5, 1.5, and 2.0 *μ*M, for 72 h. Medium was changed every 24 h and then cells were collected for RNA and DNA extraction and stored at −80°C [24].

### 2.4. Quantitative Real Time-PCR

2 *μ*g RNA of ear fibroblast cell was used to be transformed to cDNA. 0.5 *μ*L of cDNA was performed for quantitative real time-PCR with Rotor-Gene 6000 (Corbett). *β*-actin was the internal control for normalize target gene,* OPN*. The calculated gene expression fold from CT value was according to the previous study. *P* value less than 0.5 exhibited the obviously significant difference.

### 2.5. Methylation Analysis by Combined Bisulfite Restriction Analysis (COBRA)

For amplification of porcine* OPN* promoter methylation analysis site, PCR was performed using 2 *μ*L of bisulfite-converted genomic DNA as template. The primer sets of COBRA were* OPN*-C sense 5′-TTTTTTGAGGGAGATTAGTTTTTG-3′ and antisense 5′-ATTCTACTAAAATCCAACCACCC-3′. The COBRA-PCR products were purified by phenol/chloroform, followed by ethanol precipitation. The DNA was resuspended in 8.5 *μ*L of distilled deionized water. Purified PCR products were then digested with 10 U* BstU*I restriction enzyme (New England Biolabs, MA, USA) at 65°C. Products were electrophoresed on 6% native acrylamide gel, stained with 200 g/mL ethidium bromide, and visualized using a Kodak 1D software.

### 2.6. Methylation Specific-PCR

Genomic DNA (0.5 *μ*g) was treated with sodium bisulfite according to the manufacture's recommendations (EZ DNA Methylation Kit; Zymo research, CA, USA) and amplified with specific primers for methylated or unmethylated DNA. The primer sets of MS-PCR were* OPN*-M sense 5′-AAGCGGGGAAGGAGTTATTACGT-3′, antisense 5′-TCCGACAAAACGAAACGATCATACA-3′,* OPN*-U sense 5′-GAAGTGGGGAAGGAGTTTATTATGT-3′, and antisense 5′-CAATAACTCCAACAAAACAAAACAATC-3′. All PCR reactions were performed on PTC 200 thermocyclers (MJ Research, MA, USA) and in 25 *μ*L volume using the PlatiumTaq DNA polymerase system (Invitrogen, CA, USA). PCR products were separated on 1.5% agarose gels. The M-set primers contained at least three CpG sites to distinguish the methylation status of investigated region. And U-set primers overlapping the M-set primers were used to amplify the unmethylated region.

### 2.7. Plasmid Constructs

A full length pig* OPN* promoter (−2615-luc) was amplified from wild-type pig heart tissue cDNA. This fragment was cloned into a luciferase fusion plasmid, pGL3-Enhancer vector (Promega), to generate p*OPN*-full-luc.* Hind*III and* Nco*I cutting sites were used for cloning. Three truncated forms of p*OPN* promoter were prepared by PCR using the p*OPN*-full-luc as a template and using synthesized oligonucleotides as follows: p*OPN*-full-luc: sense, 5′-AAGCTTGAATTCACTCGTCTTTCCTTTGAGA-3′, and antisense, 5′-CCATGGGCTGACAGCCTGGACCTCCCC-3′; −2239-luc: sense, 5′-AAGCTTCCTATAACTGTCTACGTTCATATTAGAC-3′, and antisense, 5′-CCATGGGCTGACAGCCTGGACCTCCCC-3′; −1505-luc: sense, 5′-AAGCTTAATTTTCATTTAAGTAACCAACTTTATATATC-3′, and antisense, 5′-CCATGGGCTGACAGCCTGGACCTCCCC-3′; −495-luc: sense, 5′-AAGCTTGCCTGAACAATATAGCCTTGTCGC-3′, and antisense, 5′-CCATGGGCTGACAGCCTGGACCTCCCC-3′. The sequence of constructs was confirmed by DNA sequencing. There were two-point mutation different from NCBI: one is 287A to T and the other is 957T to A.

### 2.8. Transient Transfection and Luciferase Assay

Pig ear fibroblast cells were transfected using the Lipofectamine 2000 (Invitrogen). Fibroblast cells were incubated at a density of 8 × 10^5^ cells into 35 mm diameter dishes. After 24 h when cell was adherent to the dishes, 3 *μ*g of reporter plasmid DNA was transfected for 6 h in Lipofectamine mixture (Invitrogen). 24 h after the transfection, cell lysates were collected for a luciferase assay. The luciferase activity of the cell lysates was detected by Dual-light system (Applied biosystems). The activity data was measured with PARADIGM Detection Platforms (Beckman Coulter). Luciferase activity was normalized with 1 *μ*g *β*-gal plasmid. All luciferase assays were carried out in triplicate.

### 2.9. *In Vitro* Methylation of the* OPN* Promoter Region

The* OPN* reporter construct −495-luc was methylated by incubation with* Sss*I methyltransferase (New England BioLabs). The −2615-luc construct was methylated by* Hha*I and* Hpa*II methyltransferase (New England BioLabs) for 16 h at 37°C. The methylation status was also verified by digested with* Hha*I and* Hpa*II enzyme.

### 2.10. Electrophoresis Mobile Shift Assays

Nuclear extracts were prepared from HEK293T cells. Two probes were designed for methylation binding activity test. Two probes containing either 6–8 CpG sites or 13th CpG site in the −2615 to −2239 of the* OPN* promoter were generated by annealing two complementary oligonucleotides (*OPN 6–8*: 5′-TGCATGATCGTTCCGTCCTGCCGGAGTCACTGACGGAACCAGACCGAGGT-3′; 5′-ACCTCGGTCTGGTTCCGTCAGTGACTCCGGCAGGACGGAACGATCATGCA-3′, the predicted core sequence of the AP1 binding site is underlined;* OPN*13th: 5′-CCTCCGTGTTCCCTGTTAATGTGTAGCGCGTCGTTGTTGGGAAATAGTTC-3′; 5′-GAACTATTTCCCAACAACGACGCGCTACACATTAACAGGGAACACGGAGG-3′; the predicted core sequence of the ADR1 binding site is underlined). The transcription factor prediction software is TFSEARCH 3.0 version. The probes were labeled with *γ*-^32^P-ATP by using T4 kinase (Promega). Annealing probes also were methylated with SssI methyltransferase (NEB). Nuclear extracts containing 5.6 *μ*g of the protein were preincubated in 20 *μ*L of binding buffer (50 mM Tris-HCl (pH 8.0), 750 mM KCl, 2.5 mM EDTA, 0.5% Triton-X 100, 62.5% glycerol (v/v), and 1 mM DTT) with or without unlabeled competitor (10-fold molar excess). For supershift assay, antibody of AP1 was added to the preincubation buffer. After 10 min of preincubation on ice, the DNA probe labeled with [*γ*-^32^P]-ATP was added, and the mixtures were incubated at room temperature for 30 min. The reaction mixtures were resolved on 6% polyacrylamide gels. The gels were dried and subjected to PhosphorImager analysis using a Typhoon system and ImageQuant TL software (Amersham Biosciences, Sunnyvale, CA, USA).

### 2.11. Molecular Modelling

The molecular docking was then further analyzed for proving the further mechanism of our findings; we further surveyed the interaction of AP1 (c-Jun) and TFIIB by computational biology. The AP1 (c-Jun) is possible higher spot for hypermethylation in* OPN* promoter area and provides the binding domain for RNA-polymerase II initial binding transcription factor (TFIIB) in this study. Therefore, we first utilized the Z-DOCK program to simulate the structures of c-Jun and TFIIB. After that, we further used molecular dynamics (MD) to validate the stability of c-Jun and TFIIB complex under the GROMACS 4.5.5 program [25] with charmm27 force field. The model is set in the TIP3P water modeling in 1.2 nm distance of box for water box setting. Na and Cl ions in the concentration of 0.145 M NaCl model are used for system neutralization. All bonds are fixed by linear constraint solver (LINCS) algorithm to constrain all bonds lengths in the simulation system. Newton's Law is utilized for calculating the motion of molecular dynamics as follows:

(1) $$\begin{array}{c} \frac{d^{2}r}{dt}=M^{-1}F. \end{array}$$

The Particle mesh Ewald (PME) is also used for calculate the coulomb type of electrostatics. The Van der Waals (VDW) interactions are set as 1.4 nm cut-off distance for nonbound interaction. The first step is set on the 5,000 cycle steps performed in the manner of Steepest Descent algorithm for energy minimization. And then, equilibration was performed with a time period of 1 ns for position restraints set under the constant temperature dynamics (NVT type) conditions. The third step is calculating the production run for 5000 ps under constant pressure and temperature dynamics (NPT type). All the MD systems are set by 310 K temperature during all simulation times. MD frames data were saved every 20 ps for all production runs.

### 2.12. Molecular Dynamics Analysis

First we survey the stability of all atoms performed by using the GROMACS 4.5.5 software though the commands of g_rms and g_gyrate to calculate root mean square deviation (RMSD) and radius of gyration (Rg), respectively. Secondly, we calculate the total energy for all the systems by the command of g_energy. Thirdly, we further calculate root mean squared fluctuation (RMSF) for each protein residue by commands of g_rmsf. Fourthly, the distance between c-Jun and TFIIB and movement analysis are calculated by the g_dist program. Fifthly, the migration of dock protein (c-Jun and TFIIB) is presented by mean square displacement (MSD) under the command of g_msd module in GROMACS during all the simulation times. Sixthly, the g_cluster program is selected for further calculation of the representative structure from all MD frames, and the representative structure is taken for further snapshot analysis. We also employed DSSP analysis and matrices of the smallest distances between each residue to investigate the stability of the protein structure. The principle component analysis (PCA) is then applied to observe the protein motion changes during all the MD frames. Finally, in order to observe the compactness between c-Jun and TFIIB, Caver 3.0 software [26] was used to predicted space in the complex.

## 3. Results

### 3.1. Distribution of Porcine* OPN* CpG Island

There are dense CpG sites existing in the front of the* OPN* promoter region. One putative CpG island was found (CpG island size > 100, GC Percent > 50.0, Obs/Exp > 0.6) by MethPrimer program.

### 3.2. Methylation and Expression Analysis of* OPN* in Cloned Pig Various Tissues

**Figure 1:**
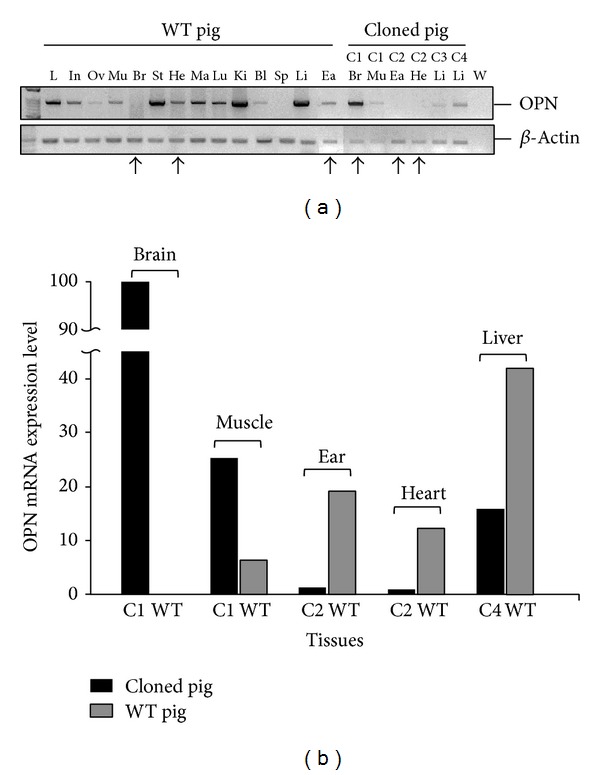
(a) (b) The semiquantitative RNA expression of* OPN*. C1–C4 indicated four different cloned pigs. The black arrows represent the contrary expression in the cloned pig tissues relative to WT tissues. Br: brain, Ea: ear, He: heart, Ki: kidney, Li: liver, Lu: lung, Mu: muscle, Sk: skin, In: intestine, Sp: spleen, Pl: placenta, Um: umbilical cord, B: blood; S: blood treated with* Sss*I, and W: ddH_2_O.

**Figure 2:**
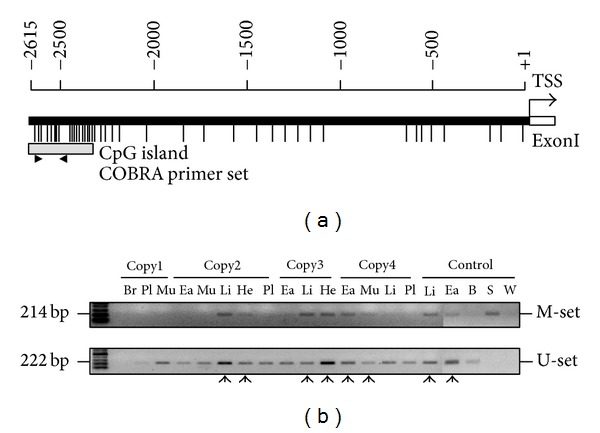
MS-PCR analysis of* OPN* promoter. (a) The distribution of CpG sites in the pig* OPN* promoter. The two arrows indicated the MS-PCR primer sets. (b) The MS-PCR results of* OPN* promoter in cloned pigs. The arrows represent both existence of methylation and unmethylation DNA element. Br: brain, Ea: ear, He: heart, Li: liver, Lu: lung, Mu: muscle, Pl: placenta, B: blood; S: blood treated with* Sss*I, and W: ddH_2_O.

Firstly, the* OPN* mRNA expression was investigated in the WT pig tissues and cloned pig tissues. Data showed the various expression levels in different tissues. Particularly, Copy1 brain overexpressed the* OPN* and Copy2 ear with no expression of* OPN* relative to its wild-type tissue, respectively (Figure 1). The unique aberrant expression patterns exhibited the different control way of the* OPN* expression. We proposed that* OPN* expression may be a tissue-specific manner. MS-PCR primers were designed to estimate the methylation status of* OPN* promoter. Hypomethylation generally appeared in the various tissues of cloned pigs. However, there were still some tissues that showed the methylated region in* OPN* promoter (Figure 2).

### 3.3. 5-aza-dc Increases* OPN* mRNA and Decreases Methylation of* OPN* Promoter in Pig Ear Fibroblast Cell

**Figure 3:**
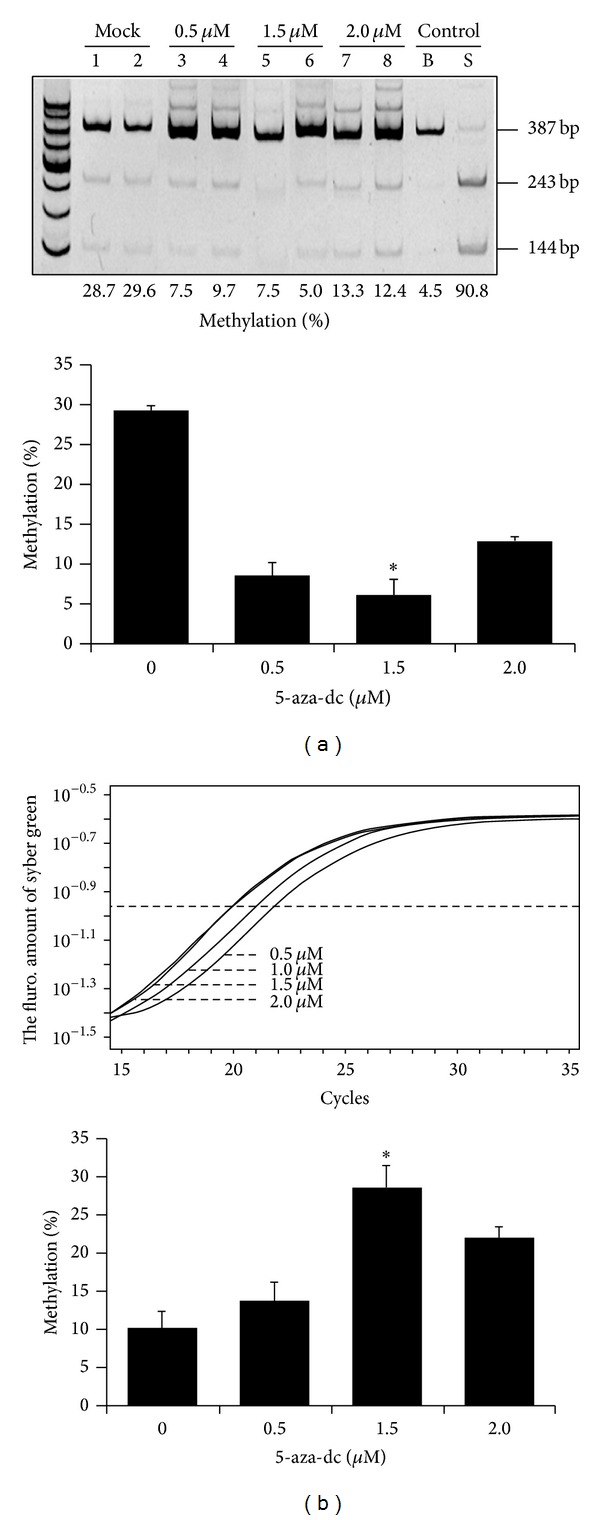
*OPN* RNA expression and DNA methylation of 5-aza-dc treated pig fibroblast cell. (a) The schematic showed the methylation percentage and mRNA expression of* OPN* promoter in different concentrations 5-aza-dc. (b) The COBRA analysis of* OPN* promoter. Below the square is the methylation percentage of* OPN* promoter methylation.

In order to realize whether the methylated* OPN* promoter affects the activity of* OPN* promoter. The 5-aza-dc treated porcine ear fibroblast cells showed that when the concentration of 5-aza-dc level increased, it will decrease the methylation of* OPN* promoter and restore the* OPN* RNA expression at 0.5 to 2.0 *μ*M (Figure 3). The results suggested that the activity of* OPN* promoter can be affected by DNA methylation directly or indirectly. COBRA assay was also used to investigate the methylation status of* OPN* promoter in WT tissues. Brain, ear, liver, and lung tissues exhibited little part methylation of the* OPN* promoter (data not show). This is thought that the methylation of* OPN* promoter in the aforementioned tissues may be involved with* OPN* transcript regulation mechanism.

### 3.4. Bisulfite Sequencing Analysis of the Whole CpG Sites Methylation Profile in Cloned Pigs

**Figure 4:**
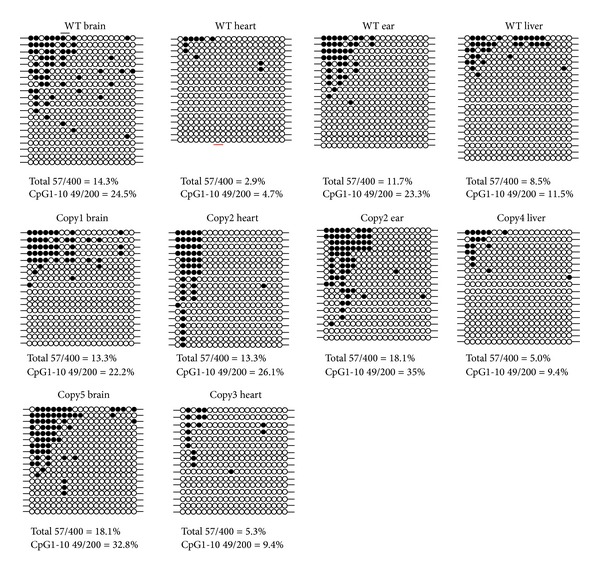
The bisulfite sequencing of* OPN* −2610~  −2400 nt upstream the promoter in cloned pigs' tissues. The closed circles represent the methylation CpG sites. The hollow circles represent the unmethylated CpG sites. The bottom number indicated the methylation percentage of each sample. The range of square showed that the region may be the methylation controlled region of* OPN* promoter.

To investigate which region of the* OPN* promoter is affected by methylation in the CpG site, bisulfite sequencing was performed to dissect the methylation status of CpG sites in the* OPN* promoter. In the brain tissue, bisulfite sequencing of Copy5 and Copy1 brain exhibited the saturated status in their methylated region while WT brain exhibited fragmentary methylated CpG sites. The 18.06% methylation percentage of Copy5 brain was more than the WT brain, 14.25% (Figure 4). In the heart tissues, bisulfite sequencing showed that the Copy2 heart had extremely hypermethylated percentage with 13.33% more than WT heart, 2.94% (Figure 4). Particularly, the data showed the inhibition of Copy2 heart mRNA (Figure 1). In the liver, the different methylation pattern also appeared in the Copy4 liver relative to WT liver. Copy2 ear with 18.05% methylation in the* OPN* analyzed region was higher than the WT ear, 11.67%; the result was proved by the experiment of* OPN* mRNA expression (Figure 1). These bisulfite sequencing results matched our previous hypothesis that methylation in the* OPN* promoter region regulates the activity of* OPN* promoter. Moreover, the hypermethylated* OPN* promoter may directly affect the activity of* OPN* transcription, especially in heart and ear tissues.

### 3.5. Analysis of Methylation Implication of* OPN* Transcription by Promoter Assay

**Figure 5:**
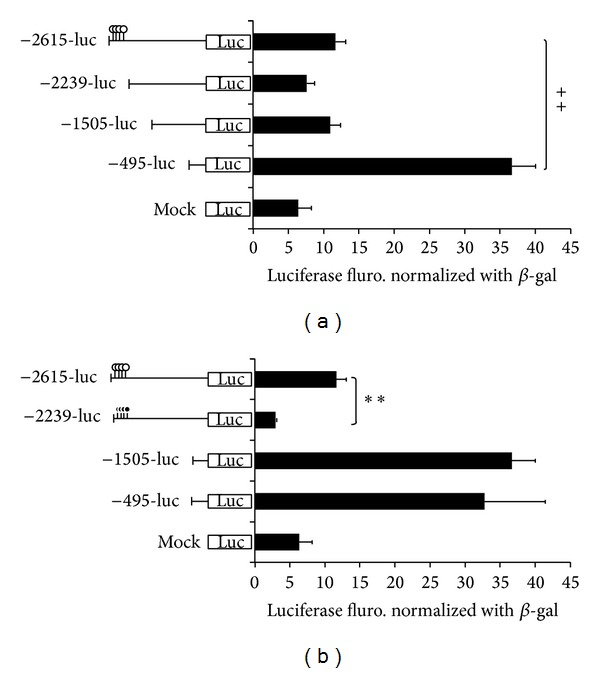
Methylation and deletion analysis of* OPN* promoter in 293T cells. The match-like bar with black circle represents the methylation CpG site; white circle of match bar indicated the unmethylation CpG sites. PGL3 vector as standard; in 293T cell line; PGL3-enhance vector as negative control; cell lysate as the background; pCMV-b-gal as internal control. The relative value is adjusted by cell lysate; −495 M-luc indicated the methylation in PGL3 backbone with* Hha*I and* Hpa*II methyltransferase. (**P < 0.01); −2615-luc and −2615 M-luc: *n* = 3; −495-luc and −495 M-luc: *n* = 4. The experiments were repeated three times and the results were analyzed and presented as the mean ± SE.

The analyzed* OPN *promoter region may involved with methylated control of gene transcription. Promoter assay was designed to explain the direct inhibition of* OPN* promoter activity by methylation on the* OPN* CpG sites. Four different truncated forms of* OPN* full length (2.6 kb) were used to prove the hypothesis (Figure 5). Particularly, the truncated form 2.2 kb deleted the 377 bp promoter region (−2615~  −2239 nt). This region is the analyzed region for bisulfite sequencing profile. And this region is thought to be the most possible element that regulates the* OPN* transcription. Moreover, four CpG sites were* in vitro* added to the methyl group by methyltransferase that can provide important evidence how methylation affect the* OPN* transcription.  Figure 5(b) shows that the* OPN* promoter activity was significantly decreased in methylated −2615-luc plasmid. However, −2239-luc that deleted the 377 bp containing methylated characteristic DNA element leads to less inhibition of promoter activity than the methylated vector −2615 M-luc (Figures 5(a) and 5(b)). The results indicated that methylation in the front* OPN* promoter is not only decreasing the promoter activity to the basal level but also recruiting the inhibition factors to enhance the inhibition ability. In order to avoid the effects of CpG sites in PGL3-enhancer backbone, −495 M-luc that have no methylated CpG sites in the* OPN* promoter but it can be methylated in the vector backbone CpG sites compared to −495-luc. Data showed that there is no difference in the promoter activity between methylation or unmethylation in the PGL3-enhancer backbone CpG sites (Figure 5(b)). It is suggested that methylation in the critical region, such as* OPN* promoter front end, may lead to the rearrangement of chromatin structure. Otherwise, deletion of the* OPN* promoter to the 495 bp with significant promotion of the promoter activity indicated that in the middle part of promoter DNA element may able to inhibit the activity of* OPN* promoter.

### 3.6. Methylation in CpG 13th and CpG 1st of* OPN* Promoter Blocks the Binding Access of Transcription Factors

**Figure 6:**
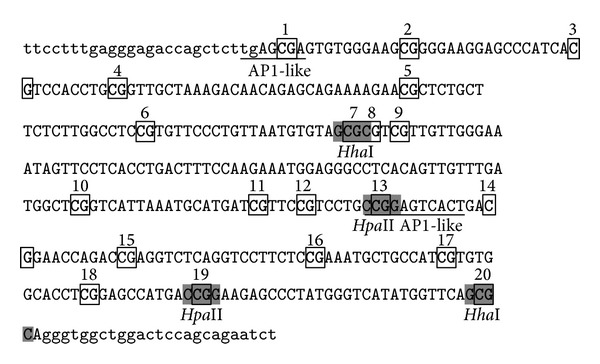
The sequence and CpG sties distribution of pig* OPN* promoter. 20 CpG sites exist in the front region of* OPN* promoter. The underline indicates the predicted AP1-like binding site. The gray marker indicates two methyltransferase sites of* Hha*I (GCGC) and* Hpa*II (CCGG).

**Figure 7:**
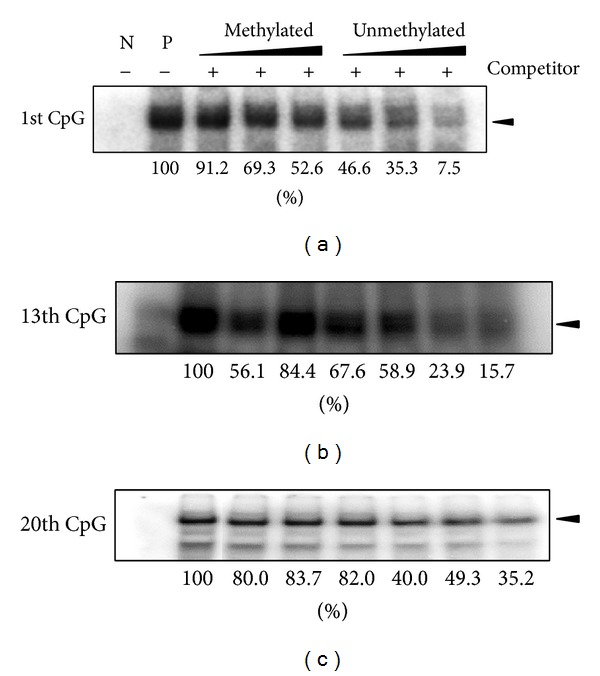
Electrophoresis mobile shift assay in porcine* OPN* promoter CpG sites. (a) Using CpG1 contained DNA element binding with SH-SY5Y nuclear extract. Methylated and unmethylated competitors were used as 2X, 5X, and 10X concentration than isotope labeled probe. (b) CpG 13th probe of* OPN* promoter binding with HEK-293 nuclear extract. (c) CpG 20th of* OPN* promoter element binding with HEK-293 nuclear extract. There was no difference between methylated or unmethylated competitors in competition experiment.

**Figure 8:**
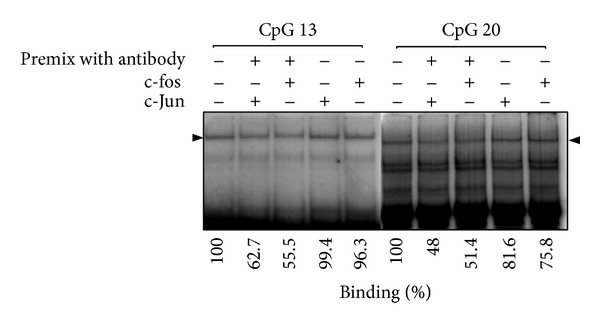
The CpG 13 and CpG 20 showed competition binding activity characteristics with c-Jun and c-Fos. The competition reduced the binding activity between transcription factors and labeled* OPN* EMSA probe. The amount of EMSA c-Jun and c-Fos antibody is 3*μ*g. The EMSA probe is 30*μ*g. Adding the Ab with a premix way can reduce the binding effects with the transcription factors. It is suggested that the premix with antibody blocks the access to its binding site (antibody-transcription factor-DNA).

We investigate that the methylated CpG sites in the −2615~−2239 nt of the* OPN* promoter region affect the transcription factor binding activity. Electrophoresis mobile shift assay was performed with nuclear extracts from human HEK293T and SH-SY5Y cell line. Four EMSA probes that contain the CpG 1, CpG 3-4, CpG 6–8, CpG 11–15, and CpG 19-20 were designed according to the CpG sites in our analyzed region (Figure 6). EMSA data suggested that CpG 13th and 1st sites showed methylation noncompetition phenomenon which had influence on binding with transcription factor (Figure 7). 19-20th CpG sites containing EMSA probe showed no competition ability in the methylated or unmethylated status. The premix with antibody and nuclear extracts by EMSA assay indicated that the c-Jun and c-Fos were involved in the binding to CpG sites 13 and 20 (Figure 8). However, the adding of antibody in the mixture of probe and nuclear extracts showed no significant shift bands. It is indicated that other transcription factors may also participate in the transcription activity* OPN* promoter. Thus, the c-Jun and c-Fos could be involved in the partial* OPN* transcriptional activity in a competition way. In addition, the c-Jun had higher binding affinity than c-Fos in the EMSA probe analysis. We therefore did followed computational survey to see why c-Juc affects the consequent* OPN* transcription.

### 3.7. The Computational Biology Results

**Figure 9:**
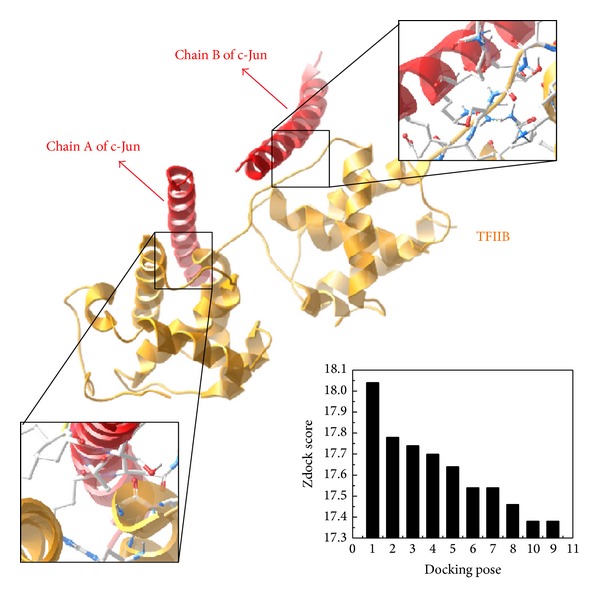
The best docking pose of c-Jun and TFIIB with 18.04 Zdock Score. The structures of c-Jun and TFIIB are colored in red and orange, respectively.

After surveying the possible zone for* OPN* promoter hypermethylation, we found that the AP1 (c-Jun) sequence frequently appeared in our molecular laboratory study. Then we further validated that the c-Jun methylation will cease the further mRNA production by inhibiting the binding of RNA-polymerase II initiation factor TFIIB. We do the further computational modelling for mechanism survey between the c-Jun and TFIIB. By the Z-DOCK analysis, we found the c-Jun and TFIIB could combine tightly (Figure 9). We chose the highest docking pose (dock score = 18.04) for further MD analysis.

**Figure 10:**
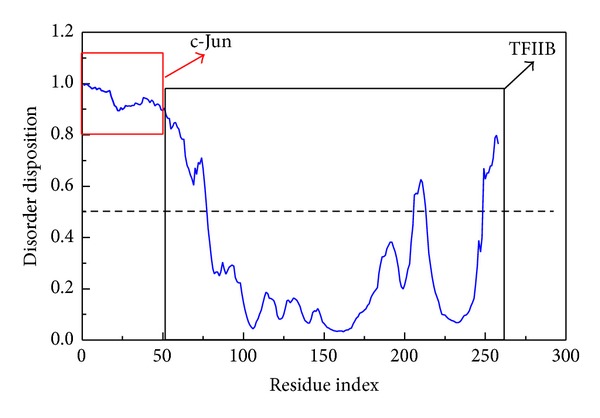
Disorder prediction of c-Jun and TFIIB complex; the value of disorder disposition below 0.5 indicates order folding region. The sequence of c-Jun is in the region from residue index 0 to 54, and the sequence of TFIIB is in the region from residue index 55 to 258. The folded structure of c-Jun reveals disorder; the N-terminal and C-terminal of TFIIB structure display folded disorder.

Then the disorder predication was employed to observe the protein folding analysis and the result is shown in Figure 10. We found that the c-Jun has relative high disorder in folding than TFIIB; we suppose that the flexibility of c-Jun structure could easier bound to TFIIB. This finding could be an explanation why the c-Jun bound to TFIIB by Z-DOCK program. To confirm the stability of the c-Jun and TFIIB complex, the series of molecular dynamic studies further visualize their interactions.

**Figure 11:**
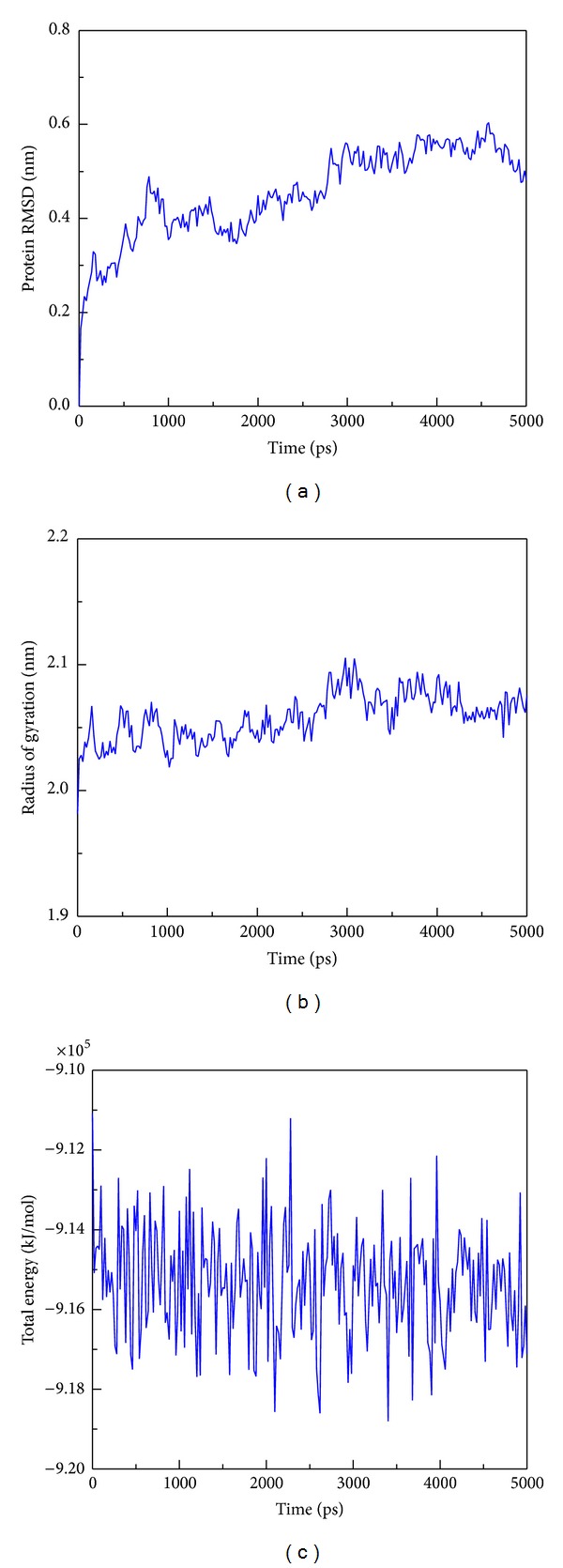
The trajectory analysis of c-Jun and TFIIB during 5000 ps simulation times. (a) RMSD values of all atoms of c-Jun and TFIIB complex; (b) radius of gyration of c-Jun and TFIIB complex for identifying the compactness of protein structure; (c) total energy of all simulated systems of c-Jun and TFIIB complex; the total energy is sum of potential energy and kinetic energy.

Protein complex RMSD analysis proved that the c-Jun and TFIIB were stable from 3000 ps to 5000 ps. In addition, we found the TFIIB are easier to be stable during the molecular dynamics (Figure 11(a)). We also found the radius of gyration tend to be stable for all simulation times with average of 2.05 nm (Figure 11(b)), suggesting that the two protein structures are compact after binding together.  Figure 11(c) also shows the binding complex in a stable fluctuation and the energy of the binding complex is stable around −9.15 × 10^5^ (kJ/mol).

### 3.8. Stability Analysis of Residues on the Major Binding Region during MD Simulation

**Figure 12:**
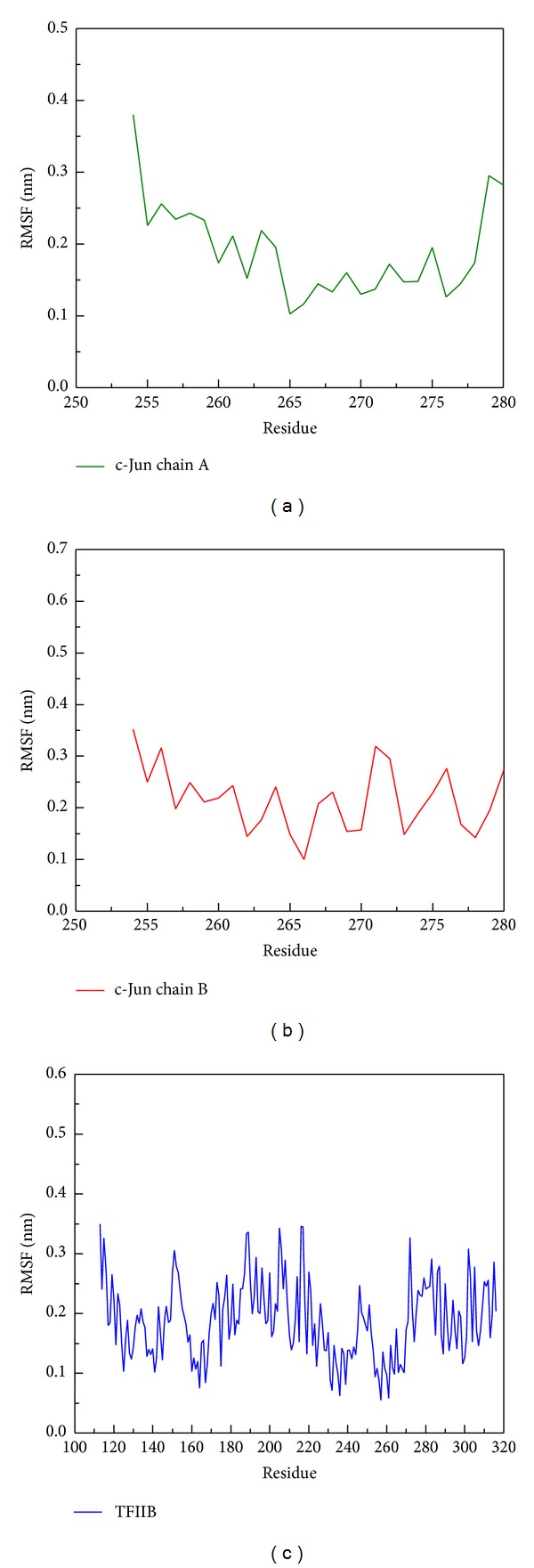
RMSF analysis of protein resides on (a) chain A of c-Jun, (b) chain B of c-Jun, and (c) TFIIB during simulation time of 5000 ps. The residue index of chain A and chain B of c-Jun is from 254 to 280, and the residue index of TFIIB is from 113 to 316. The high values of RMSF indicated the high fluctuation of residue during all simulation times.

**Figure 13:**
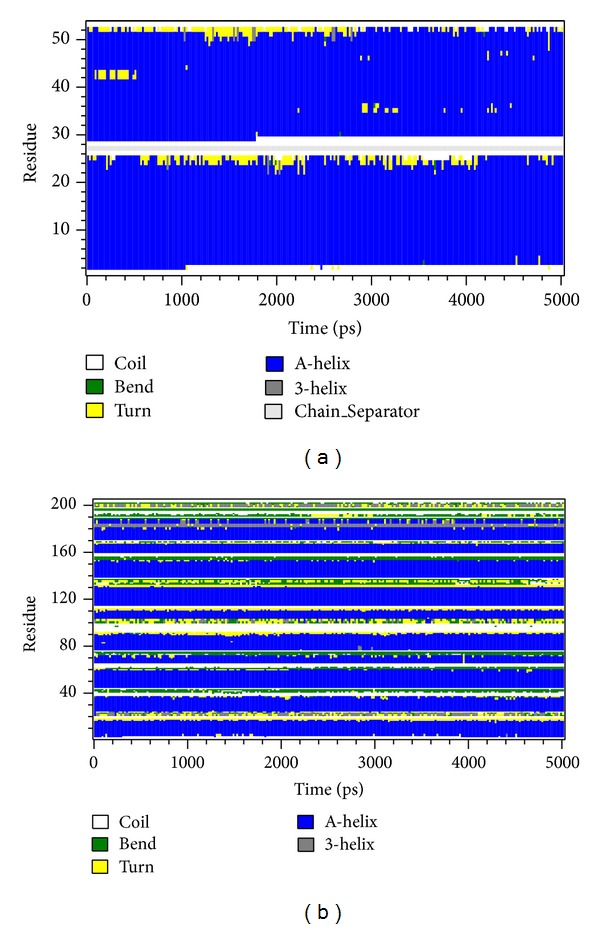
The secondary structure analysis for (a) c-Jun and (b) TFIIB over all simulation times. Each secondary type of the structure such as Bend, Turn, alpha helix (A-Helix), and 3_10_-helix (3-helix) is colored in green, yellow, blue, and gray, respectively. The “Chain_Seoarator” in (a) is used to differentiate between chain A and chain B of c-Jun.

**Figure 14:**
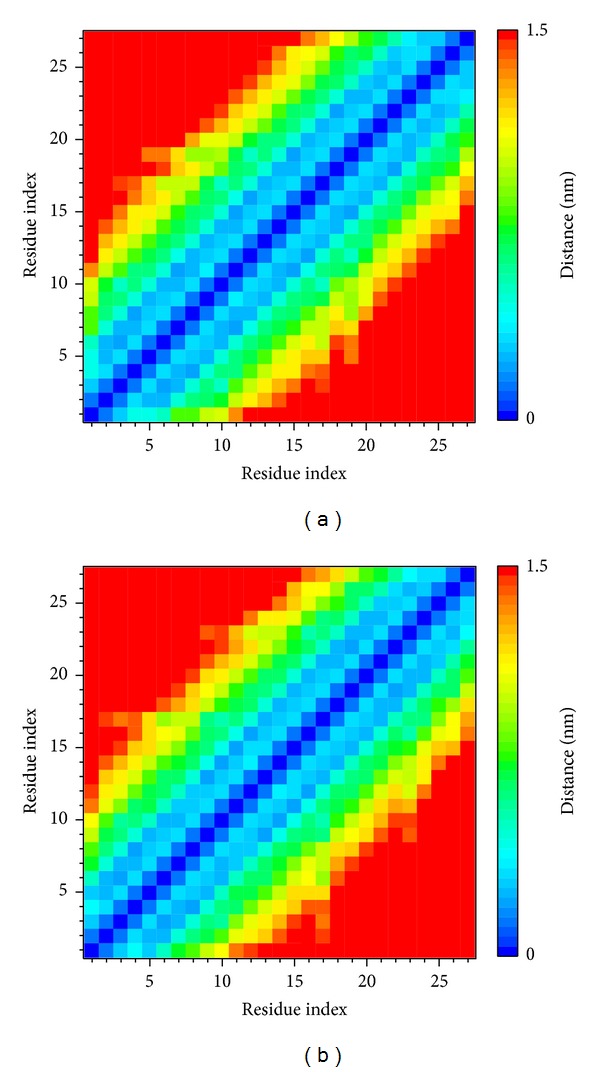
Matrices of smallest distance between each residue on (a) chain A of c-Jun and (b) chain B of c-Jun. The value of distance between residues is represented by rainbow bar, and the value of distance with longer than 1.5 nm is colored in red. The indexes of residues from 0 to 27 indicate residues from 254 to 280 on each chain of c-Jun.

To analyze the flexibility of residues on protein structure, the RMSF calculation was used to observe the flexibility of each residue; Figure 12(a) shows that the chain A of c-Jun had high frequency of fluctuation (binding site 200–210 binding resides). However, the chain B of the c-Jun has relative fewer frequency of fluctuation and the major binding region (from 228 to 240 residues) showed a less fluctuation as shown in Figure 12(b).  Figure 12(c) reveals the binding regions (200–210 binding residues) for chain A of c-Jun that is more fluctuated and unstable compared to the binding regions (228–240) for chain B of c-Jun binding site in the TFIIB binding region.  Figure 13 is the result for secondary structure variation calculated by DSSP analysis. Most of the main scaffold belong to alpha Helix; there are no significant changes during the whole MD simulation. All helices of the secondary structure for c-Jun and TFIIB binding remained stable during a 5,000 ps simulation time (Figure 13). We thereafter surveyed the distance between each residue of c-Jun for 5,000 ps. The variation of distances between residues in c-Jun chain is wider than the distances of residues in c-Jun chain B. Therefore, the chain B of c-Jun is more stable for TFIIB binding (Figure 14).

**Figure 15:**
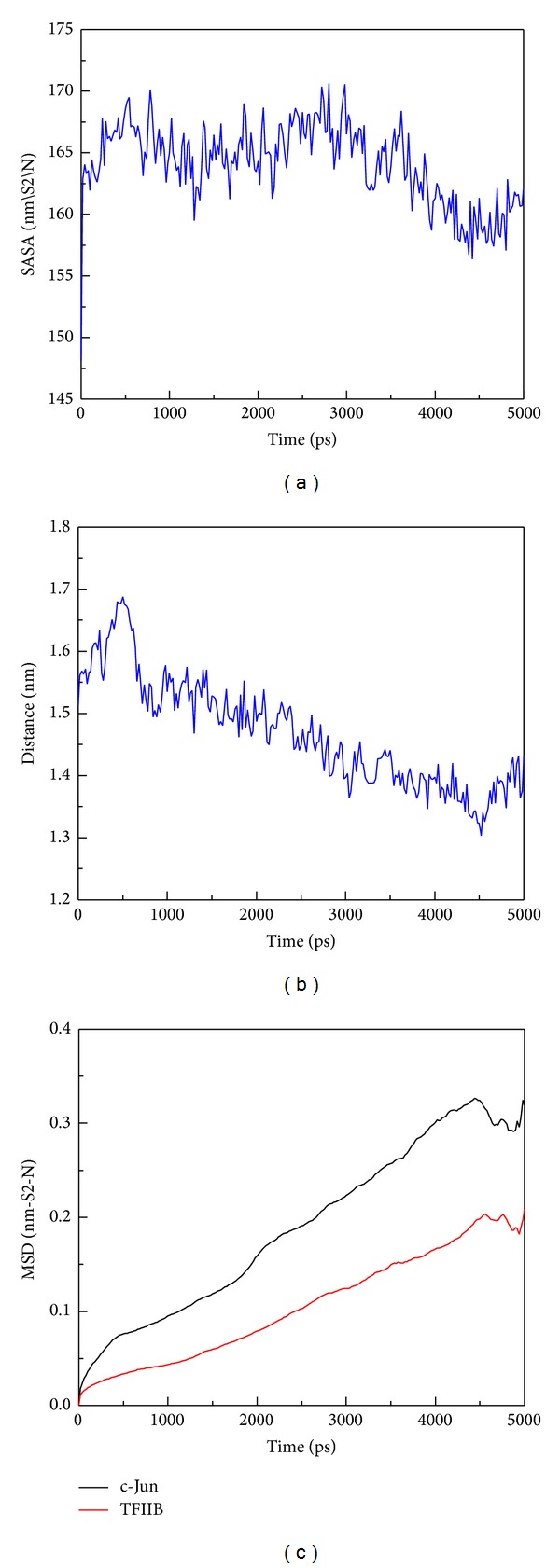
The area of solvent and protein migration analyses during simulation time of 5000 ps. (a) The total solvent accessible surface area of c-Jun and TFIIB complex; (b) the distance between the centrals of masses of of c-Jun and TFIIB; (c) trajectory analysis of MSD of c-Jun and TFIIB. The high values of MSD indicated the longer distance of migration from the initial binding position.

**Figure 16:**
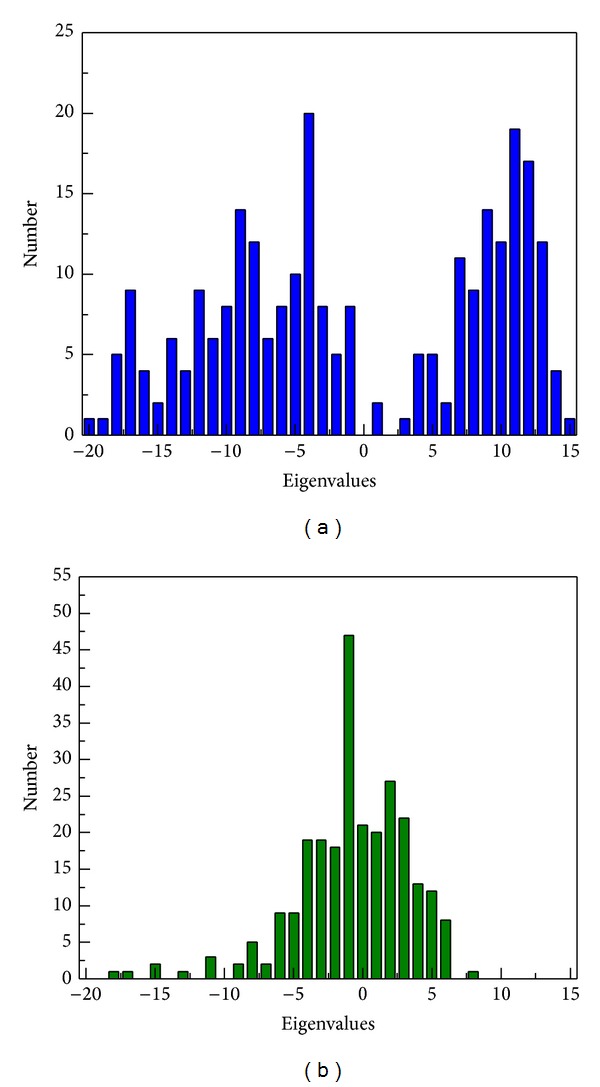
The number of MD frames of the first two eigenvectors (PC1 and PC2) by PCA analysis during simulation time of 5000 ps. The higher range of eigenvalue denotes the wider motion of protein structure over all simulation times.

**Figure 17:**
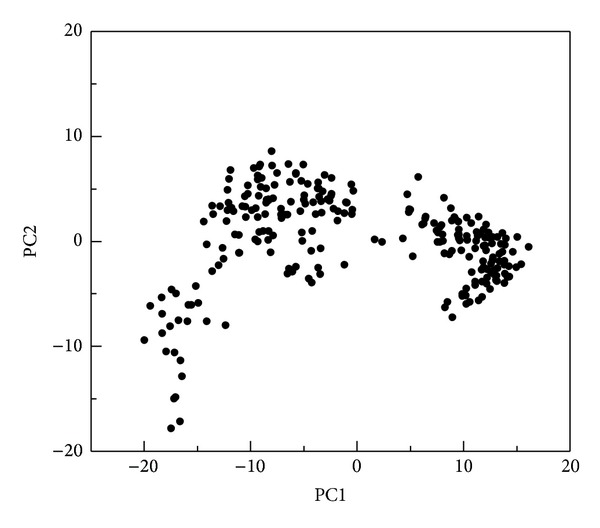
Phase space analysis of comparing the first two eigenvectors (PC1 and PC2) for principle component analysis. The eigenvalues of the two eigenvectors are projected into one phase space; small motion of protein structure could be grouped into clusters.

**Figure 18:**
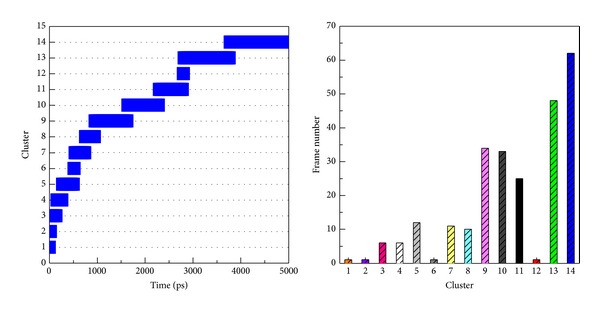
Cluster analysis of all MD frames of c-Jun and TFIIB complex during simulation time of 5 ns for identifying representative structure. All MD frames were grouped into fourteen clusters by linkage method; the RMSD cut-off distance between each neighbor frame is 0.14 nm. The most predominant group is the cluster 14, which is displaced in the region of simulation time from 4000 to 5000 ps, and the MD frames in cluster 14 are the most number among all clusters. The middle frame of cluster 14 is displaced in simulation time of 4260 ps.

**Figure 19:**
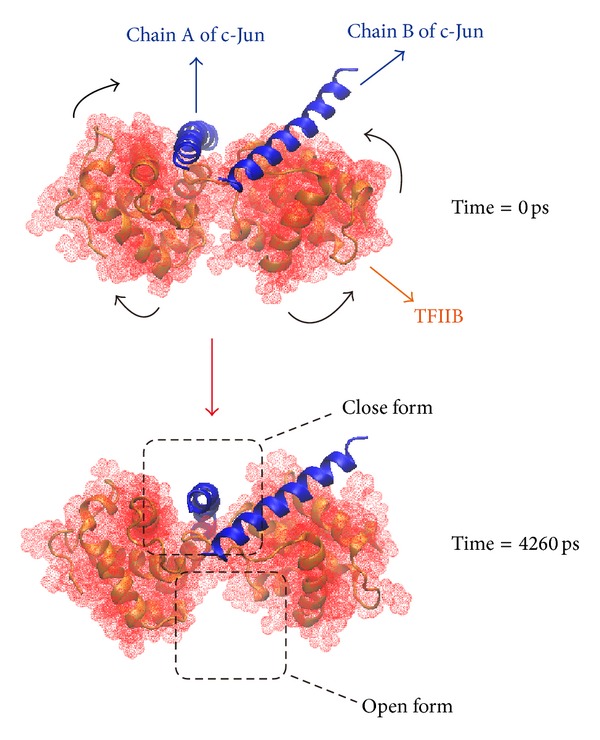
The structural comparison between the first frame (0 ps) and representative structure (4260 ps). The structures of c-Jun and TFIIB are represented by blue ribbon and red solid phase, respectively. The chain A of c-Jun was surrounded more compactly by TFIIB at 4260 ps by the structural inward rotation to make more compactness between TFIIB and c-Jun through 0 ps to 4260 ps.

**Figure 20:**
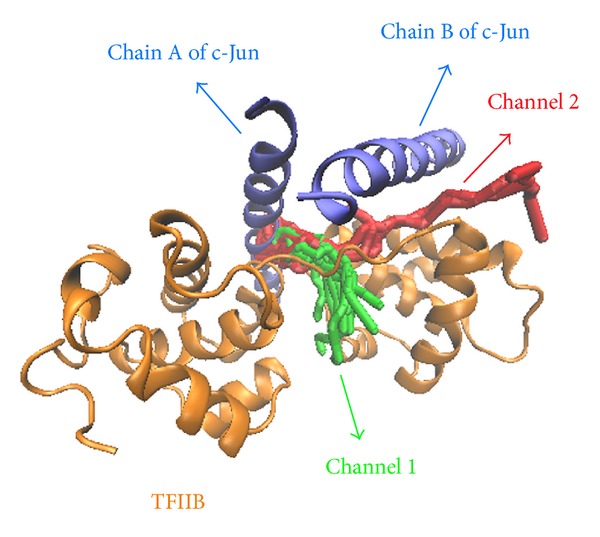
The possible space prediction between c-Jun and TFIIB among all simulation times. The predicted channels are colored in red and green. The structures of c-Jun and TFIIB are colored in blue and orange, respectively. Each possible space is represented by channels; each channel was generated by Caver 3.0 program.

The hydrophobic area was then calculated by SASA in Figure 15(a); the value of hydrophobic area decreased during the last 1000 ps. This indicated the compactness of the c-Jun and TFIIB binding increased by the MD time period in our study. It is worthy to know that the distance between centrals of masses of c-Jun and TFIIB was decreasing more and more after time goes by in the 5000 ps survey (Figure 15(b)). In the migration analysis of c-Jun and TFIIB, the MSD was employed to count the migration of c-Jun and TFIIB. The c-Jun is more unstable than TFIIB during the binding interaction throughout the whole MD simulation in 5000 ps period (Figure 15(c)). Besides, we further utilized the principal component analysis (PCA) to measure all MD frames over all simulation times. The first two eigenvectors (PC1 and PC2) were shown in Figure 18; most of frames are ranged in the short range of eigenvalues −10 and 10 in PC1 (Figure 16(a)), and arranged in eigenvalues from −5 to 5 in PC2 (Figure 16(b)). The phase space comparing for PC1 and PC2 was shown in Figure 17; we found that each frame could be grouped into two clusters. This suggests that the motion of each frame was not changed significantly over all simulation times. In order to select the most representative structure for snapshot investigation, we did cluster analysis (Figure 18). We found that the last group (cluster 14) is the predominant cluster and is also displayed in the time range from 4000 to 5000 ps; the cluster 14 also appears most predominant in frame numbers (Figure 18), and the middle structure (4260 ps) of cluster 14 is regarded as representative frame. For snapshot analysis, the comparison of initial and representative frames is shown in Figure 19; we found that the chain A of c-Jun is more encompassed by TFIIB at 4260 ps through the inward rotation of TFIIB. This made the bindings between chain A of c-Jun and TFIIB more compacted through 0 ps to 4260 ps along with time. The elevated activation between TFIIB and chain A of c-Jun is also confirmed in RMSF analysis. Therefore, we supposed the initiated transcription factor on RNA polymerase II (TFIIB) is closed interaction to the chain A of c-Jun (AP1) from 0 ps to 4260 ps. This hypothesis was also confirmed by Figure 20. There were more spaces between chain B and TFIIB than chain A. Hence, we could see that the TFIIB acts more close to the chain A of c-Jun (Figure 20). Overall, we presume that the initiation of* OPN* transcription started from TFIIB binding to chain A of c-Jun.

## 4. Discussion

Previous study has shown that DNA element (GGGTCATATGGTTCA) located in osteopontin promoter −2245 to −2259 nt can be regulated by vitamin D3 [27]. This DNA regulation region can easily be affected by the change of calcium concentration. The promoter region of porcine* OPN* was analyzed in transcription factor binding sites except the region −2615 to −2239. Interestingly, this region of porcine* OPN* promoter is rich in CpG sites compared to human, mouse, and bovine genome. Sakata also proved that* OPN* promoter transcription activity is regulated by some specific DNA modification mechanism of rearrangement of chromatin structure [22].

In the present study, four cloned pigs were surrounded by many defects. For example, Copy1 pig had a retardation of limb bone growth. Copy2 heart organ showed a pericarditis and copy3 heart had valvular heart disease. This physiology defects appeared aberrant development, especially in bone or heart, may involved in the initially fetus stage with inappropriate organ differentiate. Thus, our data suggested that the consequent result in aberrant* OPN* expression or incompletely epigenetic modification in* OPN* promoter (Figure 5). These aberrant molecular data of* OPN* are correlated with the defects of bone and heart in cloned pigs. Semiquantitative PCR of* OPN* mRNA showed that discrepant expression pattern was identified in several cloned pig tissues, especially in brain (99.75% up-regulation), heart (11.5% downregulation), and ear (18.03% downregulation) (Figure 2).* OPN* mRNA has different expression in brain development in different embryonic stages [28]. The overexpression of* OPN* in brain tissue may cause some unexpected brain damage or neuron development.* OPN* can induce myocardial fibrosis and repair tissue after inflammation. Lacking* OPN* will cause faulty wound healing after myocardial infarction [29, 30]. Silent expression of* OPN *in cloned pig' heart tissue may also be the main cause of heart disease.

Recent studies indicated that* OPN* gene expression may be affected by treatment of TSA (a histone deacetylase inhibitor). The results applied that* OPN* promoter could be regulated by epigenetic mechanism [22]. In this study, we investigated* OPN* methylation profile after 5-aza-dc treatment. The results indicated that mRNA expression of* OPN* is directly affected by adding methyltransferase inhibitor 5-aza-dc (Figure 4). COBRA was performed to study the methylation of* OPN* front end promoter in wild-type and cloned pig different tissues. Sodium bisulfite sequencing analysis also revealed that the methylation of CpG sites concentrated in front of the 20 CpG sites in front of* OPN* promoter (Figure 5). Discrepancy methylation in this promoter region also happened in brain, heart, ear, and liver tissues between wild-type and cloned pigs (Figure 5). It revealed that DNA methylation of* OPN* promoter may be involved with regulation of expression of* OPN* mRNA. In order to characterise which promoter DNA element is important, four constructs of* OPN* promoter (−2615-luc, −2239-luc, −1505-luc, and −495-luc) were used for analysis. Obvious downregulation in methylated −2615-luc (*Hpa*II and* Hha*I methyltransferase) was observed. Compared to the deletion of this control region (~390 nt) in front of* OPN* promoter, the decreasing level of promoter activity is not as obvious as −2615 M-luc construct (Figure 7). It means that methylation in the front of* OPN* promoter caused some silent mechanism that make chromosome structure more compact or block some promotion transcription factors. The EMSA data indicated that 13th CpG site of our analyzed region could bind to AP1 transcription factor and binding activity is affected by methylation in this CpG site (Figure 7). Taken together, all these findings correlated with DNA methylation in tissue- or cell-specific gene expression.* OPN* promoter region was densely methylated in some low expression (Figures 2, 4, and 5).

Our data revealed that DNA methylation of CpG sites in* OPN* promoter was the main mechanism through specific transcription factor that makes the tissue-specific expression. In previous study, AP1-like binding site (TGAGCGA) was identified as a methylated insulator region in human blastoma cell line [31]. Analyzed region in front of porcine* OPN* promoter showed that CpG 1st binding site contained the specific binding site sequence. While in our interesting DNA regulation region range from −2615 to −2239 nt of* OPN* promoter also exhibited little block access in the competition of probe. It is suggested that CpG 1st and CpG 13th play an important role in methylation controlled mechanism to regulate gene expression. We finally utilize Z-dock program [32] to analyze the interaction between AP1 (PDB code: 1JNM) and RNA polymerase II initial transcription factor (TFIIB) (PDB code: 1VOL) [33] to see if they had stable binding. From the docking result of Z-dock (Figure 8), we proved the AP1 is significantly bound to TFIIB. We also found the AP1 can autoregulate the HDAC-1 in promoter region and lead to significant higher degrees of hypermethylation in the* OPN* promoter region and cause AP1 to be hypermethylated consequently ceasing the* OPN* mRNA expression [34].

Further mechanical studies by the computational biology also pointed out that the DNA sequence for hypermethylation of* OPN* promoter binding sites is c-Jun. The chain A of c-Jun could be encompassed more tightly by inward rotational structure change of TFIIB during the MD process (Figure 19). Therefore, we found c-Jun had crucial role for interaction of initiating transcription by RNA polymerase II. The methylation of c-Jun leads to of hyper-condense helix structural change and makes transcription termination which stops* OPN* mRNA production. Therefore, the MD docking results reconfirm the c-Jun partake the crucial roles in consequent* OPN* transcription that matches our wet laboratory studies. We suppose this will cause the problems in the embryonic development and lead to threatened conditions. Therefore, adjusting* OPN* promoter c-Jun (AP1) methylation will affect transcription binding and could be the treatment for genetic developing errors in the future.

In conclusion, aberrant methylation of porcine* OPN* gene was frequently found in different tissues of somatic nuclear transferred cloning pigs and bisulfite sequence data suggested that the* OPN* promoter region of −2615 to −2239 nt may be a crucial regulation DNA element. In pig ear fibroblast cell culture study, the demethylation of* OPN* promoter was found in dose-dependent response of 5-aza-dc treatment and followed the* OPN* mRNA reexpression. In cloned pig study, discrepant expression pattern was identified in several cloned pig tissues, especially in brain (99.75% up-regulation), heart (11.5% down-regulation), and ear (18.03% downregulation). Promoter assay data revealed that four methylated CpG sites presenting in the −2615 to −2239 nt region cause significant downregulation (approximately 75%) of* OPN* promoter activity (*P* < 0.001). EMSA data also suggested that CpG 13th and 1st sites showed methylation noncompetition phenomenon which had influence on binding with transcription factor.
